# Astrovirus Diagnostics

**DOI:** 10.3390/v9010010

**Published:** 2017-01-13

**Authors:** Philippe Pérot, Marc Lecuit, Marc Eloit

**Affiliations:** 1 Institut Pasteur, Biology of Infection Unit, Inserm U1117, Laboratory of Pathogen Discovery, 75015 Paris, France; philippe.perot@pasteur.fr (P.P.); marc.lecuit@pasteur.fr (M.L.); 2 Institut Pasteur, Centre d’innovation et de Recherche Technologique (Citech), 75015 Paris, France; 3 Paris Descartes University, Sorbonne Paris Cité, 75005, Paris, France; 4 Necker-Enfants Malades University Hospital, Division of Infectious Diseases and Tropical Medicine, 75015 Paris, France; 5 Ecole Nationale Vétérinaire d’Alfort, 94700 Maisons-Alfort, France

**Keywords:** astrovirus, virology, detection methods, molecular diagnostics

## Abstract

Various methods exist to detect an astrovirus infection. Current methods include electron microscopy (EM), cell culture, immunoassays, polymerase chain reaction (PCR) and various other molecular approaches that can be applied in the context of diagnostic or in surveillance studies. With the advent of metagenomics, novel human astrovirus (HAstV) strains have been found in immunocompromised individuals in association with central nervous system (CNS) infections. This work reviews the past and current methods for astrovirus detection and their uses in both research laboratories and for medical diagnostic purposes.

## 1. Introduction

Astroviruses were first discovered in 2008 by electron microscopy (EM) examination of stool samples from children with diarrhea [1]. The virus name was given due to the star-shaped morphology of the virus, which is observed on the surface of some of the particles. Before the development of molecular techniques, EM was the only tool for laboratory diagnostics, as no cell line permissive for a broad range of strains was identified, precluding routine virus isolation. EM, and later polymerase chain reaction (PCR), increasingly demonstrated the role of astroviruses in diarrheal human disease in babies and infants (as well as in numerous animal species such as birds and mammals), and most of the population has demonstrated exposure to the virus, as is evidenced by antibody detection [2,3,4,5,6,7]. Recently, unbiased high throughput sequencing (HTS) has identified the unexpected role of astroviruses from a specific clade in human [8,9,10,11,12] and bovine [13] encephalitis. This paper summarizes the current tools available for the identification of astroviruses in diagnostic or research applications.

## 2. Virus Characteristics

Astroviruses are non-enveloped, positive sense, single-stranded RNA viruses, with solid capsid shell ~35 nm in diameter (~44 nm with spikes) [14], classified into two genera: mammalian viruses (*Mamastroviruses* (*MAstVs*), 19 species recognized by the International Committee for Taxonomy of Viruses (ICTV)) and avian viruses (*Avastroviruses* (*AAstVs*), three species recognized by ICTV). The taxonomy does not take into account the species of origin anymore. The genome is 6.8 kb to 7.9 kb in length and harbors a 5′ untranslated region (UTR), followed by three open reading frames (ORFs), namely ORF1a, ORF1b, and ORF2, a 3′ UTR and a polyA tail [15].

Human astroviruses (HAstVs) are found in four *MAstV* species (*MAstV 1, 6, 8, 9*), as summarized in Table 1. *MAstV 1* includes the eight serotypes of classic HAstVs (HAstV 1–8), a common cause of viral gastroenteritis in children, targeted by usual diagnostic PCRs. *MAstV 1* and *MAstV 6* form a monophyletic group, together with astroviruses from cats, pigs, dogs, rabbits, California sea lions, and dolphins. The two other genotypes, *MAstV 8* and *MAstV 9*, are closely related to astroviruses from mink, sheep, California sea lions, bats, cattle, pigs, and mice.

Within genotypes, strains can be grouped by serotypes, based on their antigenicity, although variability is still high, even within the same serotype. For this reason, subtypes are defined, which are well-documented within the eight serotypes of *MAstV 1*, and most likely also exist for the other species as well. Two species defined by prototypal MLB1 (*MAstV 6*) and VA1 (*MAstV 9*) strains have been described [16,17] and more recently associated to severe cases of encephalitis in immunocompromised patients [8,9,10,11,18]. High genetic variability (even within each genotype) and concerns regarding capabilities for cross-species transmission [19] are challenges for the definition of adequate diagnostic tools capable of identifying distant strains. Moreover, association between astrovirus infection and disease is still being investigated, as well as the interaction of astroviruses with other enteric viruses in diarrhea physiopathology, all of which suggests that diagnostic tools will need to continue to evolve in the near future.

## 3. Clinical Aspects of Astrovirus Infection

HAstVs are a classic cause of viral diarrhea in children, along with rotavirus, norovirus, sapovirus and adenovirus. Seroprevalence studies indicate that most children in Europe encounter astrovirus before the age of two [2]. Astrovirus-associated diarrhea is not reported in immunocompetent adults, as infection in childhood is considered to confer protective immunity. Additionally, humoral immunity is considered to play a major protective role, along with cellular adaptive immunity [20]. Therefore, immunosuppressed patients and the elderly can also develop astrovirus-associated diarrhea.

In non-immunocompromised individuals, after an incubation period of 4–5 days, an astrovirus infection will induce a mild disease, characterized by mild and short watery diarrhea for two to three days, followed by nausea, vomiting, and abdominal pain, which usually resolves spontaneously. These symptoms are most often milder than a rotavirus infection [21]. Recent seroprevalence studies have indicated that some infections can be asymptomatic as well [22]. As reported for rotavirus and norovirus, astrovirus has also been associated with intussusception in infants [23]. Although virological diagnosis of astrovirus-associated diarrhea is not routinely used in medical practice, it is sometimes used in epidemiological studies in the context of diarrheal outbreaks [24] and surveillance of diarrheal diseases [25,26].

An astrovirus infection in immunocompromised individuals may induce gastroenteritis, but it can also lead to severe and sometimes fatal systemic and central nervous system (CNS) infections, as seen in multiple cases of astrovirus-associated encephalitis and meningitis [8,9,10,11,18]. These reports are associated with newly identified HAstVs that belong to novel species (*MAstV 6* and *9*). Studies are under way to assess the actual disease burden associated with these novel neurotropic astroviruses in humans. These novel astroviruses are enteric viruses, associated with diarrhea and fecal carriage, but their pathogenicity in the non-immunosuppressed host has not yet been precisely determined, although a case of meningitis in an apparently healthy adult has recently been reported [27]. Therefore, these newly-discovered viruses seem to share some clinical characteristics with enteroviruses, due to their association with diarrhea, but may also induce meningitis and encephalitis in the immunosuppressed patients. For this reason, their detection should now be part of the laboratory diagnostic work-up in patients, in particular those who are immunosuppressed and are diagnosed with meningitis or encephalitis of unknown cause.

In mammals, astroviruses have been reported in piglets, minks and dogs with preweaning diarrhea, but accurate diagnosis is complicated due to the prevalence of fecal shedding in healthy animals, which complicates the interpretation of the results [28]. Therefore, etiological diagnostic is not a routine practice. In mink presenting with the so called “shaking mink syndrome”, and cattle with encephalitis, astroviruses can be tested in necropsy brain samples [29,30]. Additionally, astroviruses have been associated with severe avian diseases (i.e., chicken diarrhea, duck hepatitis, turkey enteritis, and avian nephritis), and diagnosis can be made in severely affected flocks by reverse transcription polymerase chain reaction (RT-PCR), using necropsy samples in specialized laboratories [15].

## 4. Methods for Virus Identification

### 4.1. Electron Microscopy (EM)

In 1975, the first observation by EM of 28–30 nm particles was reported in the stool of babies with gastroenteritis [31]. The star-shaped surface configuration of the viruses rapidly led the author to propose the name of “astrovirus” (derived from the Greek “astron” which means “star”) [32] and, since then, this morphological characteristic has been widely used for the detection of astrovirus infection in both humans and animals [33,34,35]. Direct EM is complicated by the fact that only a minority of virions exhibit a complete star-shaped structure, and careful searching may be necessary to distinguish between, for example, astrovirus and calicivirus [36], which are similar in size. Sensitivity of EM is also dependent on elevated concentrations of particles, usually around 10^7^ per gram of stool [37]. The use of immune electron microscopy (IEM) techniques using specific antibodies or convalescent sera can improve the sensitivity of the detection [38] and help with the typing [39] or the detection of new viral agents [40]. Due to the limitations described above, the use of EM for the diagnosis of viral infections has been superseded by molecular methods, and therefore, it is rarely available or used in clinical laboratories anymore.

### 4.2. Virus Isolation

Astroviruses, like other enteric viruses, can be difficult to propagate in conventional cell cultures. The first propagation of HAstVs was made possible by Lee and Kurtz in human embryo kidney (HEK) cells through the use of serum medium supplemented in trypsin [41]. After six passages in HEK, the adapted virus has been established in the continuous rhesus monkey kidney cells (LLC-MK2) and in primary baboon kidney cells, but in the absence of cytopathic effect (CPE). A 15 amino acid (aa) deletion in the non-structural polyprotein 1A may be responsible for this adaptation [42]. Other cell lines, such as African green monkey kidney Vero cells (e.g., MA-104), were not permissive for the virus even after initial passages in HEK cells [41]. However, similar experiments based on virus adaptation in embryonic kidney cells in the presence of trypsin have enabled the propagation of bovine [43] and porcine [44] astroviruses.

A major advance was made with the ability to grow *MAstV 1* in the colonic carcinoma cells (CaCo-2) directly from feces, without prior adaptation to cell culture [45]. In these conditions, a cytopathic effect is generally observed after 2–3 days of infection. A study assessing the ability of laboratory strains of HAstVs 1–7 to replicate in various human and simian cells showed that propagation can be successful in many different cells lines, including CaCo-2 and MA-104 [46]. Additionally, although adenocarcinoma cell lines appear to be the most commonly used cells to grow wild-type HAstV today, propagation directly from stool specimens is also possible in human hepatoma cell line PLC/PRF/5 [47]. While virus isolation can serve as a useful tool to investigate astrovirus biology, it is still not an ideal diagnostic tool for detection of astrovirus in diagnostic laboratories, due to slow turnaround times and difficulty of isolation.

### 4.3. Immunodetection and Antigenic Typing

The ability to grow astroviruses has simplified the production of antisera in experimental animals, allowing the characterization of serotypes [48] and the development of a radioimmune assay for detection of anti-*MAstV 1* (serotypes HAstV 1–8) antibodies [49]. Further measurement of neutralizing antibodies [50] and the production of astrovirus-specific monoclonal antibodies [51,52,53] have followed. In 1990, the first evaluation of an indirect enzyme immunoassay (EIA) that used both a monoclonal antibody directed toward the capsid of *MAstV 1* for the capture, and a polyclonal antibody for the detection, was achieved in a cohort of patients with gastroenteritis, showing a sensitivity of 91% and a specificity of 96% when compared to IEM [54]. Initially based on a peroxidase-labeled goat antibody, the detection system has been adapted with biotin-avidin, demonstrating similar performances, and was proved to be useful for large epidemiological studies and routine screening of fecal samples [55]. Of note, Moe et al. also developed an RNA-probe hybridization and tested it in parallel to their rapid biotin-avidin EIA, but although the sensitivity of the probe assay was high, it did not detect more astrovirus-positive fecal specimens than EIA [55].

Many examples of the use of EIA for clinical purposes have been observed. For instance, studies on serotype identification and prevalence in the United Kingdom [39,56] and South America [57], and efforts in typing specimen collected from several continents [58], have contributed to a better understanding of astrovirus epidemiology. More recently, rapid immunochromatography tests detecting astroviruses and claiming good sensitivity and specificity have been commercialized by several companies, but studies evaluating their performances are currently limited [59]. Although EIA tests are much easier to implement than EM and proved to be as efficient [34], their use and development have been hampered by the advent of molecular diagnostic techniques.

### 4.4. Molecular Diagnostics

#### 4.4.1. Reverse Transcription Polymerase Chain Reaction (RT-PCR) and Quantitative Reverse Transcription PCR (RT-qPCR)

Molecular approaches based on the amplification of viral genome or transcripts have dramatically improved the sensitivity of detection in comparison to EM, immunoassays or virus isolation, making substantial gains. With thresholds of detection as low as 10 to 100 genome copies per gram of stool [37,60], and the ability to develop type-specific detection systems, RT-PCR has now become a very common tool for the diagnosis of astrovirus infection in clinical laboratories. However, the design of amplification systems, in particular the intrinsic properties of the primers, are key, especially with regard to the amplification efficiencies and the ability to detect variant strains. For example, among the RT-PCR systems that have been developed for the detection of *MAstV 1* (serotypes HAstV 1–8), some are targeting non-coding regions of the virus in a very sensitive and specific manner, while others are designed into conserved motives of the capsid, thereby allowing subsequent typing but with a risk of sub-optimal amplification efficiencies (for a complete review including a table of the most commonly used RT-PCR systems, see [61]).

Alternative to RT-PCR, nucleic acid sequence-based amplification (NASBA) has also shown a good concordance with RT-PCR-based methods for the detection of *MAstV 1* (serotypes HAstV 1–8) [62]. After the discovery of distant HAstV strains, MLB [16,63] and VA1/HMO-C [17], additional primers have been developed and used to describe new populations of viruses [64,65,66,67]. Beyond human astroviruses, many RT-PCR systems were also developed to detect astroviruses in wildlife [68,69], livestock [70,71] or pets [72,73]. Although consensus primers can detect a large number of astroviruses among both animal and human strains, there is not yet a universal pan-astrovirus RT-PCR system.

In parallel to the development of PCR primers, the application of real-time PCR (qPCR) in a diagnostic setting has improved the diagnosis of astrovirus infections by reducing the risk of false positives, allowing quantitation of viral loads and shortening the time to results (a positive or negative result is usually available within 24 h of specimen collection) [74,75,76,77]. qPCR can be done using a nucleic acid stain (typically SYBR green) followed by melting curve analysis, or by the use of a specifically designed hydrolysis probe coupled with a fluorophore (typically Taqman). One-step RT-qPCR methods have also been developed [78,79]. Further refinements have been proposed with an integrated cell culture/RT-qPCR assay that is able to detect low levels of astrovirus after an incubation of seven days or less [80], but this approach has remained essentially of interest for research purposes only. Of note, although such advances have brought the ability to detect precise quantification of viral loads, the interpretation of very low amounts of virus in relation to clinical symptoms, especially in asymptomatic individuals, is still not always easy [81].

#### 4.4.2. Multiplex RT-PCR for Enteric Pathogens Panels

To meet the need for a rapid, efficient and cost-effective diagnosis, multiplex RT-PCR panels, including astroviruses and other gastrointestinal pathogens, have been developed over time. Early attempts using either end-point [82,83] or qPCR [84] proved to be as efficient as singleplex PCR for the detection in stool samples of HAstVs, noroviruses, adenoviruses, sapoviruses and enteroviruses. In the latter, the analysis of melting curves allowed the determination of dual-infection by the formation of dual peaks, while being at least 10× more sensitive than end-point PCR [84]. Since then, several multiplex assays relying on different formats of detection have been developed [85,86,87,88,89,90,91,92,93] and used for the diagnosis of astrovirus infection in humans [94,95,96,97,98,99,100,101,102] or animals [103,104,105,106,107]. Remarkably, among commercially-available solutions, the FilmArray Gastrointestinal Panel (BioFire Diagnostics, Salt Lake City, UT, USA) allows for the simultaneous detection of 22 different enteric pathogens directly from stool specimens, with a reported sensitivity and specificity of 100% and 99.9%, respectively, for the detection of *MAstV 1* (but not *MAstV 6* and *9*), and a turnaround time of around one hour [108].

Although no systematic evaluation of the clinical benefit of the many existing panels and methods has yet been published, several studies have pointed out the advantages to streamline the diagnosis of presumptive infectious diarrhea with the use of a comprehensive multiplex PCR panel for the detection of known pathogens, and also for the detection of pathogens not requested or unable to be tested by conventional tests [109,110]. For example, the FilmArray Gastrointestinal Panel has been used to detect astrovirus infections among diarrheic patients who were initially tested negative for *Clostridium difficile* and/or rotavirus [111]. In another study, the Seeplex Diarrhoea ACE Detection (Seegene, Seoul, Korea) multiplex PCR assay was used in parallel to routine assays, to detect 11 cases of astrovirus infection among 245 stool samples from pediatric patients [112]. Other broad multiplex PCR tests, including the Luminex technology (Austin, TX, USA), have been reported to offer an unprecedented ability to diagnose gastrointestinal infections in immunocompromised patients, with assay performances comparable to the tests examined [113].

#### 4.4.3. Medium to High Density Detection Systems: Nanofluidic PCR and Microarrays

With progress made in miniaturization and the development of nanofluidic systems, it has now become possible to run qPCR in parallel to nanoliter-volume chambers, thereby reducing the cost per assay. For example, a microfluidic qPCR system based on multiple singleplex TaqMan qPCR assays could quantitatively detect 13 viruses, including human astroviruses, with a sensitivity as low as two copies per microliters [114]. In another more recent study, a nanofluidic qPCR assay was used for the detection and the quantification of 19 enteric viruses and demonstrated a sensitivity lower than the limit of detection of conventional RT-qPCR and digital RT-PCR by 1.6 log and 2.7 log, respectively, for the detection of human astroviruses [115]. In both studies, a preamplification step is needed to increase the amount of target molecules present in nanovolumes. Another technique is based on parallelization of specific probe/target nucleotide hybridization on microarrays and represents an alternative for the detection of several pathogens in a single assay.

The applicability of the microarray technology for the detection of enteric pathogens had been initially evaluated for the detection of astroviruses and noroviruses in a panel of archival stool samples, allowing in some cases the characterization of known genotypes, although a considerable number of the astroviruses remained untypable [116]. Two years later, a DNA oligonucleotide microarray that used specific short capture probes of 17–20 nucleotides was proposed for the detection of all eight serotypes of HAstVs [117]. Another research initiative, referred to as the Combimatrix custom microarray, broadened the scope to the detection of human astroviruses, noroviruses, adenoviruses and rotaviruses, with the use of both conserved and variable probe sequences, showing, in particular, the absence of cross-reactivity among the four viruses [118].

Finally, a more recent format of microarray was designed to detect about 100 viral species associated with gastrointestinal infections in vertebrates in order to expand the understanding of the etiology of the disease in humans and animals [119]. It is also interesting to note that, although microarrays are based on sequence homology with predefined known pathogens, the use of a Virochip allowed the identification of an astrovirus in a domestic rabbit with gastroenteritis after traditional diagnostic approaches, including virus isolation, failed to identify the pathogen [120]. Unfortunately, the somewhat complex and costly procedures of microarray experiments may limit their use in clinic.

#### 4.4.4. High Throughput Sequencing (HTS)

The advent of HTS has opened the way to metagenomics, which is the parallel sequencing and subsequent description of all nucleic acid molecules present in a sample. Specifically, it represents a group of disruptive technologies over PCR or other hypothesis-driven detection methods. With a combination of random amplification of microbial genomes or transcripts and appropriate downstream data mining, deep sequencing has the ability to provide more detailed taxonomic information than diagnostic PCRs, and may also be used for the discovery of new pathogens without any prior hypothesis [121]. As a consequence, over the last few years, the use of HTS in research laboratories has allowed a leap forward in the identification and characterization of astroviruses in several species including humans [28,120,122,123,124,125,126,127,128,129,130,131,132,133,134,135].

Although the primary purpose of many of these studies was not the diagnosis of astrovirus infection in symptomatic situations, the identification of partial or complete astrovirus genomes among complex polymicrobial flora provided valuable insight into viral diversity, pathogenesis, and emergence of astrovirus strains. Additionally, metagenomics applied to the diagnosis of diseases of unknown origin have also resulted in the discovery of neurotropic astroviruses in humans [8,9,10,11,12,18,27], cattle [136,137,138,139,140,141] and mink [29]. However, the use of HTS for routine microbiological diagnosis remains challenging due to the high associated workload and costs. While it is true that the overall cost of HTS experiments is lower compared to other methods, a significant budget per sample is still required, in particular when only a few samples have to be analyzed independently of cohorts. Overall, the ability to deliver results in a timely manner provides significant optimizations in laboratory organization and bioinformatic workflow. For these reasons, and although HTS is now standard use for pathogen discovery, its translation into medical and actionable diagnosis still remains in infancy, and is now initially used to target the most severe and life-threatening illnesses.

## 5. Conclusions

Our understanding of the implication of astrovirus infection has greatly benefited from the evolution of technologies, from initial morphological identification to the most recent advanced high throughput molecular techniques. While several methods for the detection of astroviruses are now available, the predominant method for diagnosis in a clinical setting still remains RT-qPCR. This is due to its highly sensitive and specific nature, fast turn-around times, relatively low cost compared to more advanced molecular methods, and the ability to multiplex with other targets of interest. The main limitation of RT-qPCR, as evidenced by recent metagenomics studies, is that infection with rare or novel astroviruses will not be detected.

## Figures and Tables

**Table 1:** Human astroviruses (HAstVs).

**Genus**	*Mamastrovirus (MAstV)*			
**Species**	*MAstV 1*	*MAstV 6*	*MAstV 8*	*MAstV 9*
**Serotypes and strains**	HAstV 1*	MLB 1	VA2/HMO-A	VA1/HMO-C
	HAstV 2*	MLB 2	VA4	VA3/HMO-B
	HAstV 3*	MLB 3	VA5	
	HAstV 4*		BF34	
	HAstV 5*			
	HAstV 6*			
	HAstV 7*			
	HAstV 8*			

*, classic human astroviruses.
